# CSAD Ameliorates Lipid Accumulation in High-Fat Diet-Fed Mice

**DOI:** 10.3390/ijms232415931

**Published:** 2022-12-14

**Authors:** Rongrong Tan, Jiayang Li, Lu Liu, Qian Wu, Lei Fan, Ningning Ma, Chuwei Yu, Henglei Lu, Xuemei Zhang, Jing Chen, Likun Gong, Jin Ren

**Affiliations:** 1 Department of Pharmacology, School of Pharmacy, Fudan University, 826 Zhangheng Road, Shanghai 201203, China; 2 Center for Drug Safety Evaluation and Research, State Key Laboratory of Drug Research, Shanghai Institute of Materia Medica, Chinese Academy of Sciences, 501 Haike Road, Shanghai 201203, China; 3 University of Chinese Academy of Sciences, No.19A Yuquan Road, Beijing 100049, China; 4 School of Life Science and Technology, Shanghai Tech University, 100 Haike Road, Shanghai 201210, China

**Keywords:** NAFLD, CSAD, obesity, liver damage, fatty acid β-oxidation, mitochondrial damage

## Abstract

Non-alcoholic fatty liver disease (NAFLD) is a chronic metabolic disease manifested in hepatic steatosis, inflammation, fibrosis, etc., which affects over one-quarter of the population around the world. Since no effective therapeutic drugs are available to cope with this widespread epidemic, the functional research of genes with altered expression during NAFLD helps understand the pathogenesis of this disease and the development of new potential therapeutic targets for drugs. In the current work, we discovered via the analysis of the Gene Expression Omnibus (GEO) dataset that cysteine sulfinic acid decarboxylase (CSAD) decreased significantly in NAFLD patients, which was also confirmed in multiple NAFLD mouse models (HFD-fed C57BL/6J, db/db and HFHFrHC-fed C57BL/6J mice). Next, CSAD’s function in the progression of NAFLD was explored using AAV-mediated liver-directed gene overexpression in an HFD-fed mouse model, where the overexpression of CSAD in the liver could alleviate NAFLD-associated pathologies, including body weight, liver/body weight ratio, hepatic triglyceride and total cholesterol, and the degree of steatosis. Mechanically, we found that the overexpression of CSAD could increase the expression of some genes related to fatty acid β-oxidation (Acad1, Ppara, and Acox1). Furthermore, we also detected that CSAD could improve mitochondrial injury in vitro and in vivo. Finally, we proposed that the effect of CSAD on lipid accumulation might be independent of the taurine pathway. In conclusion, we demonstrated that CSAD is involved in the development of NAFLD as a protective factor, which suggested that CSAD has the potential to become a new target for drug discovery in NAFLD.

## 1. Introduction

Non-alcoholic fatty liver disease (NAFLD) is a metabolic disease characterized by ectopic triglyceride-based lipid accumulation in the liver without a history of alcohol abuse, which appears as a spectrum of lesions ranging from fatty liver to non-alcoholic steatohepatitis (NASH), fibrosis and cirrhosis [1]. Currently, it has become one of the most prevalent diseases affecting more than one-quarter of the global population because its development and progression are closely associated with diabetes [2] and obesity. According to epidemiological studies in 2019, South America had the highest incidence (41%), followed by the Middle East (32%), Asia (27%), and Europe (23%), and this incidence is increasing year by year [3]. Although some drugs, such as several non-antidiabetic drugs [4] have been reported to show beneficial effects on NAFLD, there have been no FDA-approved drugs so far to cope with this widespread epidemic.

The pathological features of NAFLD include insulin resistance, steatosis, hepatocyte injury, inflammation, and fibrosis, which are strongly linked with ER stress, mitochondria dysfunction, cell death, etc [5]. However, the exact mechanisms underlying the occurrence and development of NAFLD are not fully understood. An increasing number of studies have revealed that NAFLD-associated pathologies are highly correlated with the alteration of gene expression profiles in the liver by RNA-seq or protein mass spectrometry [6,7]. The association of the rs495392 Klotho polymorphism with hepatic steatosis has been discovered in NAFLD patients using the MassARRAY [8]. Diego Almanza found that significantly differentially expressed genes are associated with metabolic processes and immune responses in a mouse model of diet-induced obesity using RNA-seq [9]. Meanwhile, the studies of these differentially expressed genes help explore the pathogenesis of NAFLD and provide potential targets for drug development [10,11]. For example, the expression of DUSP12 is downregulated in the liver of ob/ob mice compared with lean mice, and its overexpression reduces high-fat diet (HFD)-induced hepatic steatosis, insulin resistance, and inflammation [12].

Cysteine sulfinic acid decarboxylase (CSAD) is a key enzyme in the route of taurine production. It can convert cysteine sulfinic acid, the product of cysteine dioxygenase 1 (CDO1), to hypotaurine, which leads to the synthesis of taurine. It has been reported that the levels of taurine are downregulated in cardiomyopathy, retinal degeneration, prenatal and postnatal growth retardation, and obesity, while CSAD knockout mice exhibit altered histology of the lung, kidney, retinas, and pancreas, which indicated that taurine and CSAD are required for lung, kidney, immune function, etc [13,14,15]. Additionally, dysfunctional glucose metabolism was also discovered in Csad^-/-^ mice. But its change during NAFLD and exact effect on NAFLD has never been elucidated.

In the present study, we found that CSAD was downregulated in NAFLD patients and model mice, and then its function was explored in vitro and in vivo, providing a clue that CSAD might be an important factor in the pathogenesis of NAFLD.

## 2. Results

### 2.1. CSAD Is Downregulated in NAFLD Patients and Multiple NAFLD Mice Models

**Figure 1:**
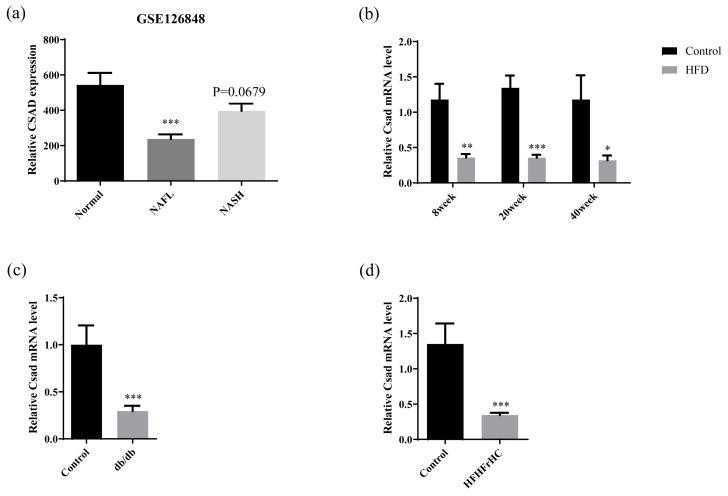
CSAD was reduced when compared with healthy controls in several NAFLD models. (**a**) The level of hepatic CSAD mRNA in the clinical database (GEO126846); n = 14 in the normal group, n = 15 in the NAFLD group, and n = 16 in the NASH group. *** *p* < 0.001 vs. normal group. (**b**) The levels of Csad mRNA in the livers of HFD mice and control mice; n = 4–6 in each group. The mice were fed with a high-fat diet (HFD group) or normal diet (control group) for 8, 20, and 40 weeks respectively. Then, the level of Csad mRNA in the liver was detected by RT-qPCR; * *p* < 0.05, ** *p* < 0.01, *** *p* < 0.001 vs. the control group. (**c**) The levels of Csad mRNA in the livers of *db*/*db* mice and WT mice; n=5 in each group. The *db*/*db* mice and WT littermates were fed a normal diet for 20 weeks. Then, the level of CSAD mRNA in the liver was detected by RT-qPCR; *** *p* < 0.001 vs. the control group. (**d**) The levels of Csad mRNA in the livers of HFHFrHC mice and control mice; n = 3–10 in each group. The mice were fed a high-fat, high-fructose, and high-cholesterol diet (HFHFrHC group) or a normal diet (control group) for 20 weeks. Then, the level of Csad mRNA in the liver was detected by RT-qPCR. *** *p* < 0.001 vs. control group.

Through analyzing the differently expressed genes during the progression of NAFLD in the GEO database (GSE126848), we found that CSAD expression was decreased significantly in NAFL and NASH patients. (Figure 1a). Next, we further investigated whether this change in CSAD occurred in the early stage of NAFLD. The Csadexpressions in the liver of HFD-fed mice for 8, 20, and 40 weeks were detected by RT-qPCR. As shown in Figure 1b, the levels of Csad mRNA in the livers of HFD-fed mice were significantly reduced compared to normal-fed mice at different time points. Interestingly, the reduction in Csad mRNA was evident even in mice with HFD feeding for 8 weeks, which suggested that Csad may play an important role in the early progression of NAFLD. Additionally, we also detected the expression of Csad in other NAFLD mouse models. Since db/db mice are frequently used as the NAFLD model [16], the hepatic Csad was examined in this mouse model. As shown in Figure 1c, the mRNA levels of Csad were reduced in db/db mice compared with WT mice. Meanwhile, the mRNA levels of Csad were still reduced in the high-fat, high-fructose, and high-cholesterol (HFHFrHC)-fed NAFLD mice model. (Figure 1d).

### 2.2. Overexpression of CSAD Improved HFD-Induced Obesity and Liver Damage

**Figure 2:**
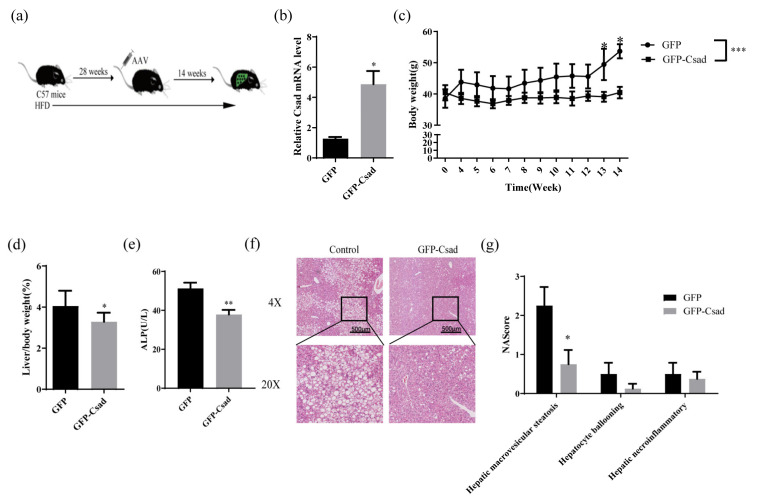
Overexpression of CSAD-inhibited weight gain and liver injury induced by an HFD. (**a**) The schematic diagram of animal experiments. (**b**) The expression of CSAD in the liver. (**c**) The body weight of mice during the whole experiment. Two-way ANOVA was used to analyze the data. *** *p* < 0.001 (**d**) The ratio of liver weight to body weight. (**e**) The content of ALP in serum. (**f**) Representative images of H&E staining of liver tissues. (**g**) The histological scores of macrovesicular steatosis, ballooning, and necroinflammatory activity for (**f**). The pathologist, who was blinded to the experimental protocol, assessed semi-quantitatively according to the NAFLD activity scoring (NAS) system. Fourteen weeks post-injection of AAV, mice were sacrificed, and the CSAD in the liver was detected by RT-qPCR, the content of ALP in serum was detected by the automatic biochemistry analyzer, and a portion of liver tissue was fixed and then stained by hematoxylin and eosin; n = 4 in GFP group, n = 8 in Csad group; * *p* < 0.05, ** *p* < 0.01vs. GFP group.

Since CSAD expression is associated with NAFLD, the biological function of CSAD in NAFLD was then investigated. AAV-mediated liver-directed gene overexpression was used to overexpress Csad in the liver of mice fed an HFD for 28 weeks (Figure 2a). After 14 weeks of overexpression, hepatic Csad mRNA was detected by RT-qPCR. As shown in Figure 2b, the Csad mRNA level was significantly upregulated in the Csad group compared to the GFP group.

The body weight in the GFP group increased gradually with the induction of the HFD, accompanied by higher levels of adipose tissue. In the Csad group, the body weight had no longer enhanced (Figure 2c). Furthermore, no significant change was observed in epididymal and perirenal fat (Figure S1a,b).

To evaluate the effect of Csad on liver injury, the liver/body ratio, serum biochemical indexes, and H&E staining of the liver were examined. As shown in Figure 2d, the liver/body ratio in the Csad group was significantly lower than that in the GFP group. At the same time, ALP in the serum of the Csad group was reduced (Figure 2e). Meanwhile, a decrease in ALT and AST in the serum was also observed in the Csad group, but without a statistical difference (Figure S1c,d). The results of liver sections stained with H&E demonstrated that the Csad group had less macrovesicular steatosis and ballooning than the GFP group (Figure 2f), which was reflected in the lower histological scores in theCsad group according to pathophysiological evaluations (Figure 2g). However, there was no difference in the grade of necroinflammatory activity between the two groups, which indicated Csad did not influence inflammation.

The above results indicated that CSAD overexpression can relieve liver injury by reducing the liver/body ratio, and ALP, and by improving liver morphology.

### 2.3. Overexpression of CSAD-Alleviated Hepatic Steatosis

**Figure 3:**
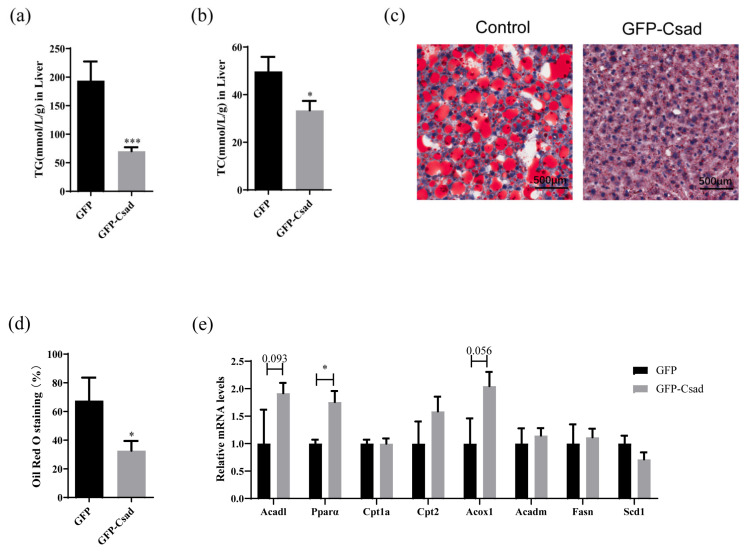
Overexpression of CSAD-reduced lipid accumulation enhanced the expression of β-oxidation-related genes in the liver. (**a**) The levels of TG in the liver. (**b**) The levels of TC in the liver. (**c**) Representative images of Oil Red O staining of liver tissues. (**d**) The quantification of Oil Red O staining of liver tissues. (**e**) The levels of *Acad1*, *Ppara*, *Cptla*, *Cpt2*, *Acox1*, *Acadm*, *Fasn,* and *Scd1* mRNA in the livers were detected by RT-qPCR. Fourteen weeks post-injection of AAV, the mice were sacrificed and the contents of TG and TC in the liver were detected using an enzymatic reagent kit, and a portion of liver tissue was frozen in OCT compound and then stained with Oil Red O solution; n = 4 in GFP group, n = 8 in Csad group; * *p* < 0.05, *** *p* < 0.001 vs. GFP group.

Hepatic steatosis occurs in the initial stage of NAFLD [17]. To investigate the effect of Csad on hepatic lipid accumulation, the levels of TC and TG in the liver were detected. In Figure 3a,b, the results indicated that the overexpression of Csad could reduce the levels of TG and TC in the liver of HFD mice. Furthermore, Oil Red O staining, a histological marker of TG accumulation, was used to confirm the function of Csad in hepatic steatosis. As shown in Figure 3c, TG accumulation in the liver was significantly lessened after the overexpression of Csad than in the GFP group. The statistical analysis of the sections showed that the ratio of Oil Red O-stained area to the liver area was significantly reduced in the Csad group (Figure 3d). Additionally, dyslipidemia also occurs in the initial stage of NAFLD [17]. To investigate the effect of Csad on dyslipidemia, the contents of TG, TC, HDL-C, and LDL-C in the serum were tested. As shown in Figure S2a–d, the content of serum TG, TC, HDL-C, and LDL-C in the Csad group was unchanged. However, the result of the OLTT showed that the Csad group can enhance lipid metabolism (Figure S2e) compared to the GFP group.

According to the above results, the overexpression of CSAD can improve hepatic lipid accumulation. Therefore, we assayed the mRNA levels of lipid metabolism-related genes in the liver, including *Acad1*, *Ppara*, *Cptla*, *Cpt2*, *Acox1*, *Acadm*, *Fasn*, and *Scd1*. As shown in Figure 3e, some genes related to fatty acid β-oxidation (*Acad1*, *Ppara*, *Acox1*) were increased after the overexpression of Csad, while there were no changes between the control and Csad group for two genes related to fatty acid and TG synthesis (*Fasn* and *Scd1*).

These results indicated that the overexpression of CSAD can alleviate hepatic steatosis in the liver, which might be related to its upregulation of the β-oxidation gene.

### 2.4. Overexpression of CSAD Can Attenuate Mitochondrial Damage

**Figure 4:**
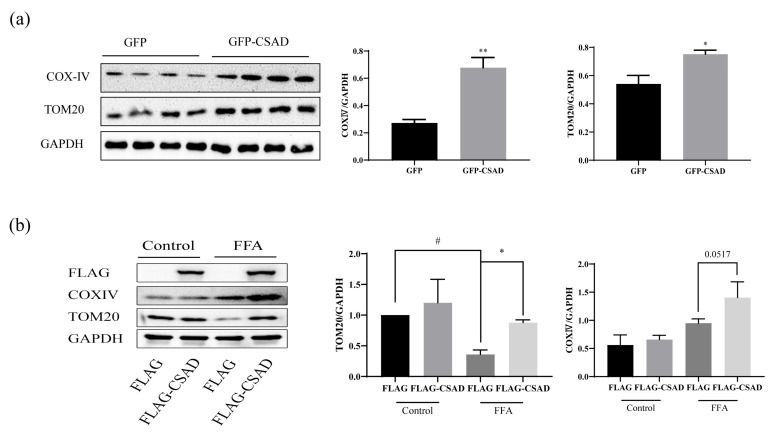
Overexpression of CSAD can attenuate mitochondrial damage in vivo and in vitro. (**a**) The levels of COX-IV and TOM20 in the liver. Fourteen weeks post-injection of AAV, the mice were sacrificed and the levels of COX-IV and TOM20 in the liver were detected by Western blot. In addition, the quantitative analysis used GAPDH as a normalizer; * *p* < 0.05, ** *p* < 0.01 vs. GFP group. (**b**) The levels of COX-IV and TOM20 in HepG2 cells. After being transfected with FLAG or FLAG-CSAD for 48 h, the HepG2 cells were incubated with or without 1mM FFA for 24 h, and the levels of TOM20 and COX-IV were detected by Western blot and quantitative analysis using GAPDH as the normalizer; # *p* < 0.001 vs. FLAG in the control group; * *p* < 0.01 vs. FLAG in FFA group.

As reported, mitochondria in hepatocytes were damaged due to lipid peroxidation and oxidative stress during the process of NAFLD [18]. The improvement of mitochondrial function could protect hepatocytes and alleviate NAFLD. Given that the overexpression of CSAD can upregulate the expression of β-oxidation-related genes in vivo (Figure 3e), we speculated that CSAD could improve the state of mitochondria to modulate lipid metabolism. As shown in Figure 4a, the overexpression of CSAD increased the protein levels of cytochrome oxidase (COX)IV, a key regulator of energy production in mitochondria, and the mitochondrial outer membrane protein TOM20 in the liver when compared with the GFP control group.

To further confirm this protective effect of CSAD on mitochondria damage in vitro, the levels of TOM20 and COX-IV were examined in HepG2 cells after being transfected with Flag-CSAD or its control for 48h and then co-incubated with free fatty acid (FFA, palmitic acid (PA): oleic acid (OA) = 1:2, 1 mM) for 24 h. As shown in Figure 4b, the levels of TOM20 were reduced due to mitochondrial damage induced by FFA treatment, whereas CSAD overexpression could remit this lesion. Although COX-IV did not decrease after FFA treatment, CSAD could further enhance its protein level, which indicated CSAD is capable of enhancing mitochondrial function. Moreover, we constructed a cell model in L02 incubated with 0.5 mM PA for 24 h, in which the level of TOM20 and COX-IV was significantly reduced due to mitochondrial damage, while the CSAD overexpression could restrain this reduction (Figure S3), which was similar to in vivo results.

**Figure 5:**
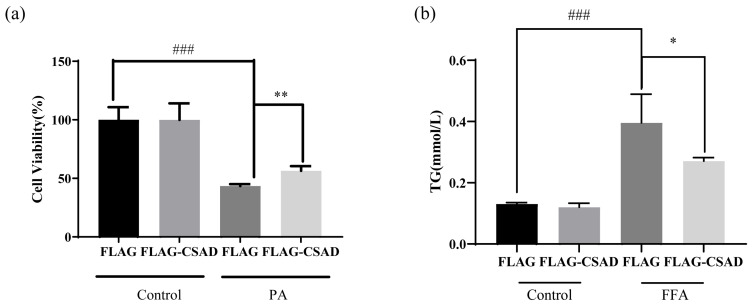
Overexpression of CSAD can ameliorate the decrease in cell viability and lipid accumulation caused by lipotoxicity in HepG2 cells. (**a**) The cell viability of HepG2 cells. After being transfected with FLAG or FLAG-CSAD for 48 h, the HepG2 cells were incubated with or without 0.5 mM PA for 24 h, and the cell viability was detected using the CCK-8 assay; ### *p* < 0.001 vs. FLAG in the control group; ** *p* < 0.01 vs. FLAG in PA group. (**b**) The content of TG in HepG2 cells. After being transfected with FLAG or FLAG-CSAD for 48 h, the HepG2 cells were incubated with or without 1mM FFA for 24 h, and the content of serum TG was detected using an enzymatic reagent kit; ### *p* < 0.001 vs. FLAG in the control group; * *p* < 0.01 vs. FLAG in FFA group.

Based on the above results, we found that the overexpression of CSAD in vivo and in vitro can improve the mitochondrial injury of hepatocytes. Therefore, to confirm the protective effect of the overexpression of CSAD on hepatocytes in vitro, we also measured the cell viability after PA (0.25 mM, 24 h) treatment and the amount of TG after FFA (1 mM, 24 h) treatment in HepG2 cells. As shown in Figure 5a,b, CSAD overexpression could improve damaged cell viability by PA treatment and reduce the levels of TG increased by FFA treatment.

These results suggested that CSAD could protect mitochondria and further prevent cell death and lipid accumulation.

### 2.5. Overexpression of CSAD Does Not Affect the Production of Taurine

**Figure 6:**
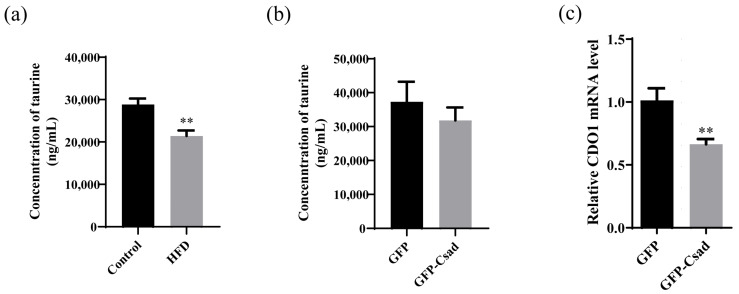
Overexpression of Csad does not influence taurine. (**a**) The content of taurine in the serum of HFD-fed mice and control mice; n = 5 in each group. The mice were fed with a high-fat diet (HFD group) or normal diet (control group) for 40 weeks, the blood was collected and the content of taurine in the serum was detected by HPLC-MS/MS; ** *p* < 0.01 vs. control group. (**b**) The content of taurine in the serum of HFD-fed mice with overexpression of GFP-Csad or GFP. Fourteen weeks post-injection of AAV, blood was collected and the content of taurine in serum was detected by HPLC-MS/MS. (**c**) The levels of CDO1 in the liver. Fourteen weeks post-injection of AAV, the mice were sacrificed and the levels of CDO1 in the liver were detected by RT-qPCR using GAPDH as the normalizer; n = 4 in the GFP group, n = 8 in the Csad group; ** *p* <0.01 vs. GFP group.

As reported, taurine can reduce mitochondrial damage during the process of NAFLD [18]. Our results showed that the overexpression of CSAD, a key enzyme in the taurine synthesis pathway in the NAFLD mouse model, can improve mitochondrial damage. To investigate whether the improvement of CSAD on mitochondrial damage is related to taurine, we measured the levels of taurine in the liver of NFALD-model mice overexpressing CSAD. As shown in Figure 6a, the content of taurine in serum was reduced in HFD mice compared with normal mice at a 40-week induction time, which is consistent with the lower expression of CSAD at this point (Figure 1b). However, the content of taurine in serum did not increase when CSAD was overexpressed (Figure 6b). Interestingly, we found that CDO1, another key enzyme in the production of taurine, was downregulated in the GFP-CSAD group (Figure 6c).

These results suggest that the protective effect of CSAD on mitochondrial damage and lipid accumulation may not be related to taurine.

## 3. Discussion

As a chronic epidemic, the pathogenesis of NAFLD is still unclear, which restricts its drug development [19]. The study of proteins that are differentially expressed during the pathogenesis of NAFLD can promote an understanding of the pathogenesis of NAFLD and provide new targets for drug development. Through analyzing differential gene expression in the liver from the GEO database (GSE126848), we found that CSAD was significantly decreased under the state of NAFLD. Next, this reduction in hepatic CSAD mRNA was confirmed by the comparison of db/db and WT mice, or the comparison of mice induced with an HF or HFHFrHC diet with those fed a normal diet. Further experimental results showed that CSAD was downregulated in HFD-fed mice, even for 6 weeks, the earliest time for high-fat-induced metabolism disorders. Combined with previously published data indicating that CSAD is reduced under western-diet stimuli [20] and CSAD gene polymorphism (rs11170445) is associated with the occurrence of type 1 diabetes through genome-wide association analysis (GWAS) [21], we believe that the downregulation of CSAD expression may be closely related to metabolism, especially for NAFLD.

The diagnosis of NAFLD is mainly divided into invasive and non-invasive measurements [22,23]. A liver biopsy is an invasive way and the most accurate diagnostic tool at present, but this method carries some small risks of serious morbidity. Non-invasive diagnostic methods are mainly imaging and biomarker testing. For example, METS-IR, an insulin resistance index, is more useful than HOMA-IR to predict the prevalence of NAFLD in middle-aged and older Korean adults [24]. However, the early stage of NAFLD is difficult to diagnose due to a mild clinical manifestation. Therefore, non-invasive diagnostic methods should be imminently developed for the early diagnosis of NAFLD. We found that the content of taurine in serum was downregulated with the decrease in CSAD expression. Since the expression of CSAD changed in the early stage of NAFLD, the change in taurine in blood might have occurred in the early stage. Meanwhile, taurine has been reported to alleviate dyslipidemia, hepatic steatosis, and insulin resistance in NAFLD animal models, which means that taurine played an important role in NAFLD [25,26]. Based on the above results, we hypothesized that the content of taurine in the blood might become a new biomarker for the early diagnosis of NAFLD, which requires further clinical validation.

The beneficial effect of CSAD in vivo was detected in our study, which was consistent with the reported function of taurine in improving NAFLD [15,27]. However, the content of taurine was not increased by CSAD overexpression in our study (Figure 6b). The reason for this is probably because CDO1, another key enzyme in the production of taurine, was downregulated in the mice model (Figure 6c). Due to the downexpression of CDO1, the content of taurine did not change in our study. Meanwhile, other research indicated that the administration of taurine to Csad^-/-^ mice does not alleviate the abnormalities in glucose metabolism caused by CSAD knockout [15]. Thus, CSAD might have other mechanisms to improve metabolic disorders, which need further experiments. Based on our results, CSAD overexpression could protect mitochondria from the damage induced by an HFD in vivo or PA in vitro. As reported, there is a close link between mitochondrial dysfunction and the progression of NAFLD [28]. Regulating mitochondrial-related proteins could improve the symptom of NAFLD. For example, PGC-1α or Mfn-2 were reduced in non-esterified fatty acids (NEFAs) induction, while their overexpression could reverse the mitochondrial damage caused by NEFAs [29]. Further, the compound targeting mitochondrial-related protein also has an improving effect on NAFLD. For example, cyanidin-3-O-glucoside (C3G) could increase PTEN-induced kinase 1 (PINK1)/Parkin expression and mitochondrial localization to suppress hepatic oxidative stress, NLR family pyrin domain containing 3 (NLRP3) inflammasome activation, steatosis and improve systemic glucose metabolism in mice with NAFLD [30]. Our results concerning the relationship between CSAD and mitochondria provide new evidence for the conclusion that protecting mitochondria may be an effective way to ameliorate NAFLD.

It has been reported that many dietary ingredients including bile acids, cholesterol, and soluble and insoluble fibers can regulate hepatic CSAD enzyme activity. Furthermore, bile acids have been confirmed as negative regulators of CSAD mRNA via regulating SHP and FXR pathways [31]. Edaravone (EDA) is widely used in the treatment of stroke. Ma et al. demonstrated that EDA can promote the expression of CSAD and speculated that CSAD might be a potential target of EDA therapy for stroke [32]. With the determination of the crystal structure of CSAD [33] and the in-depth study of its function and related mechanism, more regulatory factors will be discovered to regulate CSAD in the future, which might also provide more possibilities for the treatment of NAFLD.

## 4. Materials and Methods

### 4.1. Mice Experimental Protocol

All animal protocols were approved by the Institutional Animal Care and Use Committee of the Shanghai Institute of Materia Medica, Chinese Academy of Sciences. Mice were housed in a specific pathogen-free laboratory (23 ± 1 °C, 12 h light/12 h dark cycles, 50% relative humidity).

For the detection of hepatic Csad in multiple NAFLD mice models: (1) HFD-fed mice (2017-01-RJ-137). The male C57BL/6J mice (Shanghai Jihui Laboratory Animal Care Co., Ltd., Shanghai, China) were fed with a normal diet (24.02% of calories derived from protein; 63.03% from carbohydrate; and 12.95% from fat—Beijing KeAo XieLi feed Co., Ltd, Beijing, China) or high-fat diet (20% of calories derived from protein; 20% from carbohydrate; 60% from fat—Research Diets, New Brunswick, NJ, USA) after 1 week of acclimation. During the 8th, 20th, and 40th week of the dietary intervention, the livers of the mice were collected, respectively, and stored at −80 °C. (2) db/db mice (2018-01-RJ-160). db/db mice and WT mice aged 21 weeks (Shanghai Jihui Laboratory Animal Care Co., Ltd., China) were fed with a normal diet and were sacrificed and the liver was collected for subsequent analysis. (3) HFHFrHCD-fed mice (2021-05-RJ-241). The male C57BL/6J mice (Shanghai Jihui Laboratory Animal Care Co., Ltd., China) were fed with a normal diet or a high-fat, high-fructose, and high-cholesterol diet (71.5% Purina Rodent Chow; 0.5% Cholesterol; 5% Fructose; 11.5% Coconut Oil; and 11.5% Corn Oil by weight—Research Diets, USA) after 1 week of acclimation. In the 12th week of the dietary intervention, the livers of the mice were collected for subsequent analysis.

For the CSAD functional investigation(2019-04-RJ-191), the male C57BL/6J mice were fed an HFD as described before for 28 weeks and then infected with adeno-associated viruses (4 × 1011 viral particles per mouse) by tail vein injection, in which GPAAV-CMV-FE1-ZsGreen1-WPRE (Genomeditech, Shanghai, China) was the GFP group (n = 4), and GPAAV-CMV-mCsad-FE1-ZsGreen1-WP was the GFP-Csad group (n = 8). Then, the mice continued to be fed with an HFD until sacrificed. During the period, OLTT was tested at the end of the 10th week. Fourteen weeks post-injection of AAV, the mice were fasted overnight and sacrificed. A portion of liver tissue was fixed in 4% paraformaldehyde in PBS for hematoxylin and eosin (H&E) staining, and the rest of the liver tissue was frozen in a liquid nitrogen tank and then stored at −80 °C until used for subsequent analysis.

### 4.2. Biochemical Analysis of Blood Samples

The contents of serum alanine aminotransferase (ALT), aspartate transaminase (AST), alkaline phosphatase (ALP), low-density lipoprotein cholesterol (LDL-C), high-density lipoprotein cholesterol (HDL-C), triglyceride (TG), and total cholesterol (TC) were assayed with an automatic biochemistry analyzer from Roche (Cobas C501).

### 4.3. TG and TC Analysis

TG and TC levels of liver tissue and HepG2 cells were assayed using an enzymatic reagent kit (Nanjing Jiancheng Bioengineering Institute, Nanjing, China) according to the manufacturer’s protocol. Briefly, for tissue, a piece of liver is placed in a 1.5 mL tube and 150 µL of absolute ethanol was added. The contents were ground thoroughly using a homogenizer and centrifuged at 4 °C, 2500 rpm for 10 min. For cells, the cells in a 24-well plate were lysed by 50 µL 1% triton x-100. A 2.5 µL sample of supernatant was transferred to a 96-well plate and 250 µL of detection solution was added. Samples were incubated at 37 °C for 10 min and the absorbance was measured at 510 nm. The final concentration was normalized by the weight of the resected liver section (for tissue) or by the total protein concentration (for cells) detected by the BCA protein assay kit (Thermo Fisher Scientific, Waltham, MA, USA).

### 4.4. Oral Lipid Tolerance Tests (OLTT)

Mice were fasted overnight and orally administered olive oil (1.5 mL/kg body weight). Then, blood was collected at 0, 1, 2, 4, and 6 h from the caudal vein. The content of serum TG was assayed using an enzymatic reagent kit (Nanjing Jiancheng Bioengineering Institute) according to the manufacturer’s protocol.

### 4.5. Histologic Analysis

For H&E staining, hepatic specimens were formalin-fixed, paraffin-embedded, and sectioned. Then, H&E staining was performed with a standard protocol. Histological steatosis, ballooning, and necroinflammatory activity were assessed semi-quantitatively by a pathologist who was blinded to the experimental protocol, according to the NAFLD activity scoring (NAS) system [34].

For Oil Red O staining [35], frozen hepatic sections were conserved in OCT compound (Tissue-Tek, Torrance, CA, USA), cut into 5-μm-thick sections, washed with 60% isopropanol for 30 s, stained with Oil Red O solution (in 60% isopropanol, Sigma, St. Louis, MO, USA) for 5 min, then washed with 60% isopropanol for 30 s. Finally, tissue sections were stained with hematoxylin for 10 s and the degree of steatosis was analyzed using ImageJ.

### 4.6. RT-qPCR

Total RNA was extracted from liver tissues using Trizol reagent (Takara, Maebashi, Japan) and reverse transcribed to cDNA with PrimeScriptTM RT Master Mix (Takara) according to the manufacturer’s instructions. The RT-qPCR analysis was performed using a 7500 Fast Real-Time PCR System (7500 Software v2.0.5, Applied Biosystems, Waltham, MA, USA) using SYBR Premix Ex Taq TM (Takara). For each sample, GAPDH was used for normalization purposes. The following primers were used:

M-Csad-F: CCAGGACGTGTTTGGGATTGT;
M-Csad-R: CTCCTTCCATTCGCAGACCTT;
M-Acadl-F: TTTCCTCGGAGCATGACATTTT
M-Acadl-R: GCCAGCTTTTTCCCAGACCT
M- Ppara-F: AACATCGAGTGTCGAATATGTGG
M-Ppara-R: CCGAATAGTTCGCCGAAAGA
M-Cpt1a-F: TGGCATCATCACTGGTGTGTT
M-Cpt1a-R: GTCTAGGGTCCGATTGATCTTTG
M-Cpt2-F: CAGCACAGCATCGTACCCA
M-Cpt2-R: TCCCAATGCCGTTCTCAAAAT
M-Acox1-F: TAACTTCCTCACTCGAAGCCA
M-Acox1-R: AGTTCCATGACCCATCTCIGTC
M-Acadm-F: AGGGTTTAGTTTTGAGTTGACGG
M-Acadm-R: CCCCGCTTTTGTCATATTCCG
M-Fasn-F: GGAGGTGGTGATAGCCGGTAT
M-Fasn-R: TGGGTAATCCATAGAGCCCAG
M-Scd1-F: TTCTTGCGATACACTCTGGTGC
M-Scd1-R: CGGGATTGAATGTTCTTGTCGT
M-GAPDH-F: GACGGCCGCATCTTCTTGTG
M-GAPDH-R: GCGCCCAATACGGCCAAATC

### 4.7. Plasmid Construction

The coding sequence (CDS) of human CSAD was obtained from the cDNAs of L02 cells. The primers were designed as follows: forward primer: gacaagcttgcggccgccatgtcaattccactgaagtcctcatt; reverse primer: gtttttgttcggatcctcacaggtcctggcctagccgctc. Using the EcoR1 restriction sites, we inserted the PCR product into the p3 × FLAG-myc-CMV-24 (Sigma-Aldrich, St. Louis, MO, USA). The integrity of the CSAD was evaluated by DNA sequencing.

### 4.8. Western Blot

Mice liver tissues, HepG2 or L02 cells were lysed in lysis buffer (1M Tris, pH 7.6, 1M NaCl, 1% NP40, 5% sodium deoxycholate) with proteinase inhibitor (Thermo Fisher Scientific). Total protein lysates were quantified with Pierce BCA Protein Assay Kit (Thermo Fisher Scientific), separated by SDS-PAGE and electro-transferred to PVDF membranes (Millipore, Burlington, MA, USA), then incubated with antibodies against, COX-IV (Protintech, Rosemont, IL, USA), TOM20 (Santa Cruz Biotechnology, Dallas, TX, USA) and GAPDH (CST) overnight at 4 °C, respectively. Protein bands were visualized using a Lumi QECL regent solution kit (Share-Bio, Hangzhou, China) according to the manufacturer’s instructions. GAPDH was used as the internal control.

### 4.9. Cell Viability Assay

HepG2 cells were seeded into a 96-well plate at a density of 10,000 cells/well and cultured in a humidified incubator at 37 °C with 5% CO_2_. After being transfected with FLAG or FLAG-CSAD for 48 h, the cells were incubated with 0.5 mM PA for another 24 h. Next, 10 µL CCK8 reagent (Beyotime, Nantong, China) was added to each well and the plate was placed in the incubator for 1 h at 37 °C. Finally, the optical density value (OD450) was measured.

### 4.10. Taurine Content Analysis

The content of taurine in mice serum was detected by HPLC-MS/MS (AnLing Biomed (Suzhou) Co., Ltd., Suzhou, China). Briefly, take 20 µL mice serum, add 20 µL water and 180 µL acetonitrile, then vortex. Centrifuge at 4 °C, 4000× *g* rpm for 10 min. Take 100 µL supernatant and add 100 µL water for dilution. Then, 190 µL acetonitrile/water (1/1, *v*/*v*) was added to a 10 µL diluted sample and then vortexed to determine.

### 4.11. Statistical Analysis

The Student’s *t*-test was used to measure the differences between the two groups, and statistical significance was assessed by *p*-value thresholds: * *p* < 0.05; ** *p* < 0.01; *** *p* < 0.001. The data in vivo were given as mean ± SEM. The data in vitro were given as mean ± SD.

## 5. Conclusions

In the present study, we found that CSAD is downregulated in the NAFLD model, and further results have shown that CSAD overexpression can alleviate NAFLD-associated pathologies, including body weight, liver/body weight ratio, hepatic triglyceride and total cholesterol, and the degree of steatosis, probably by improving mitochondrial injury in HFD mice. Based on the above results, we expounded on the role of CSAD in the pathogenesis of NAFLD disease and provided a clue that CSAD might be a new drug target for NAFLD.

## Data Availability

The data presented in this study are available on request from the corresponding author.

## Supplementary Materials

The following supporting information can be downloaded at: https://www.mdpi.com/article/10.3390/ijms232415931/s1.

## Abbreviations

NAFLD	non-alcoholic fatty liver disease
CSAD	cysteine sulfinic acid decarboxylase
GEO	gene Expression Omnibus
NASH	non-alcoholic steatohepatitis
ALT	alanine aminotransferase
AST	aspartate transaminase
LDL-C	low-density lipoprotein cholesterol
HDL-C	high-density lipoprotein cholesterol
TG	triglyceride
TC	total cholesterol
COX-IV	cytochrome c oxidase subunit 4
TOM20	translocase of outer mitochondrial membrane 20
GAPDH	glyceraldehyde-3-phosphate dehydrogenase;
Acad1	acetyl-CoA acetyltransferase 1
Ppara	peroxisome proliferator-activated receptor α
Cptla	carnitine palmitoyl transferase 1a
Cpt2	carnitine palmitoyltransferase 2
Acox1	acyl-CoA oxidase 1
Acadm	acyl-CoA dehydrogenase
Fasn	fatty acid synthase
Scd1	stearyl coenzyme A desaturation enzyme 1
