# Supplementary material for: CSAD Ameliorates Lipid Accumulation in High-Fat Diet-Fed Mice
Source: Int J Mol Sci. 2022 Dec 14;23(24):15931. doi: 10.3390/ijms232415931 (PMC9783087; doi:10.3390/ijms232415931)
Supplement: Supplementary file 1 [file ijms-23-15931-s001.zip › ijms-2050006-supplementary.pdf]

International Journal of
Molecular Sciences

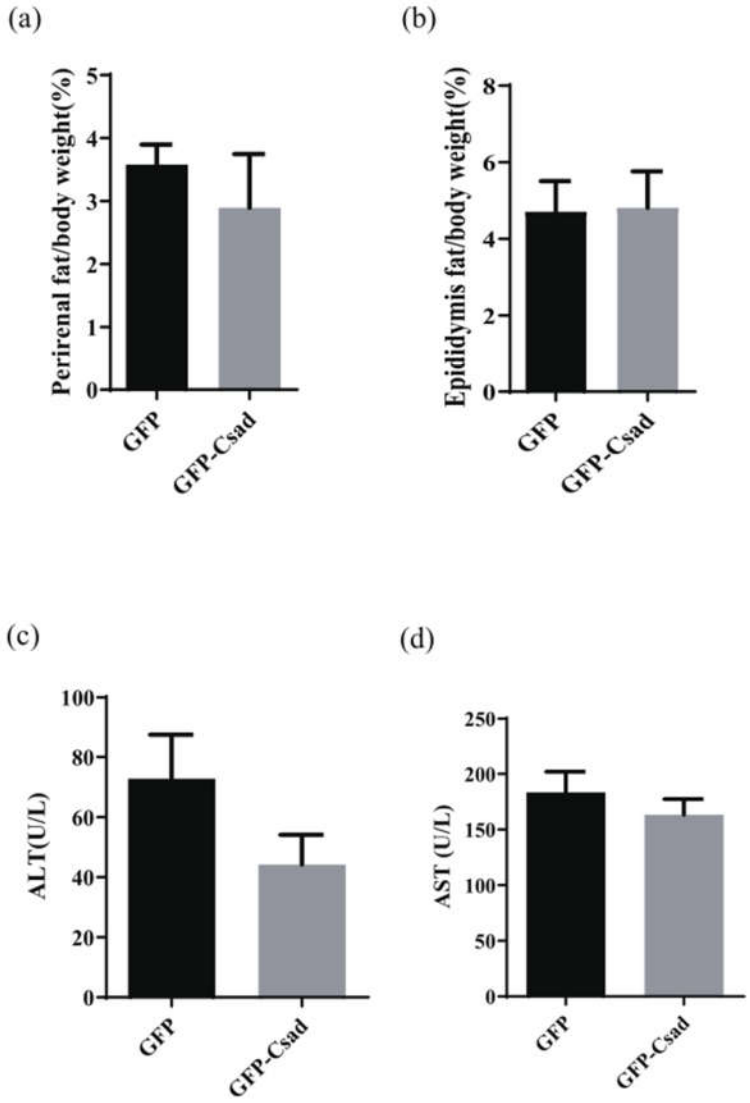

Supplementary Figure S1. Effect of Csad on visceral fat, serum ALT and AST in HFD-fed mice. The ratio of perirenal fat (**a**) or epididymal fat(**b**) weight to body weight. The content of ALT(**c**) and AST(**d**) in serum. Fourteen weeks post injection of AAV, mice were fasted overnight and sacrificed. The weight of perirenal fat, epididymal fat and body were recorded. The blood was collected and the contents of ALT and AST in serum were detected by the automatic biochemistry analyzer. n=4 in GFP group, n=8 in Csad group.

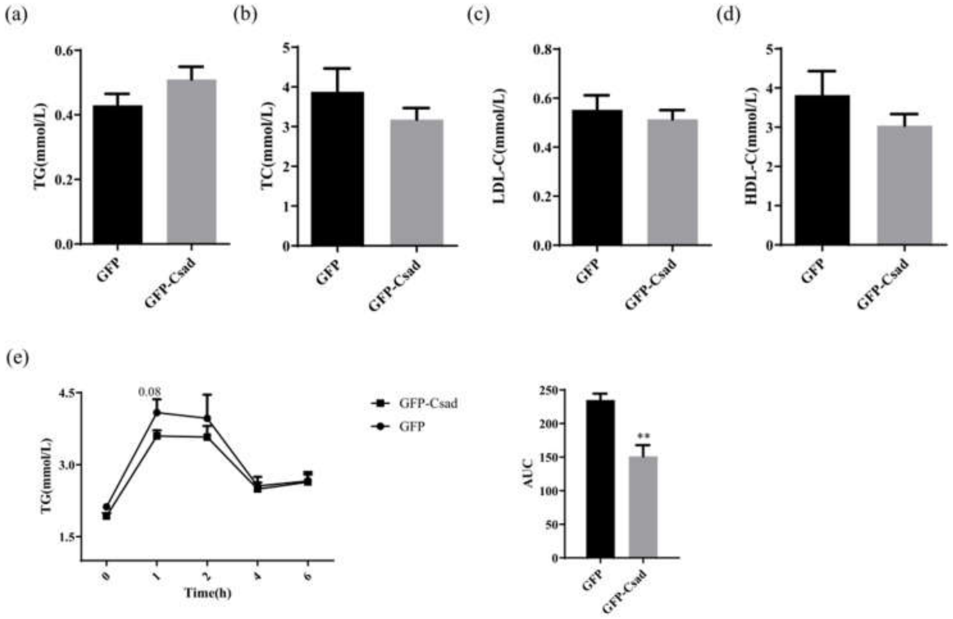

Supplementary Figure S2. Effect of Csad on serum lipid in HFD-fed mice. The levels of TG(**a**), TC(**b**), LDL-C(**c**), and HDL-C(**d**) in serum. Fourteen weeks post injection of AAV, the blood was collected and the contents of TG, TC, LDL-C and HDL-C in serum were detected by the automatic biochemistry analyzer. (**e**) The content of serum TG and corresponding AUC in OLTT assay. After oral administration of olive oil (1.5 ml/kg body weight), the blood was collected at 0, 1, 2, 4, 6h and the content of serum TG was detected by enzymatic reagent kit. n=4 in GFP group, n=8 in Csad group. **P<0.01*vs* GFP group.

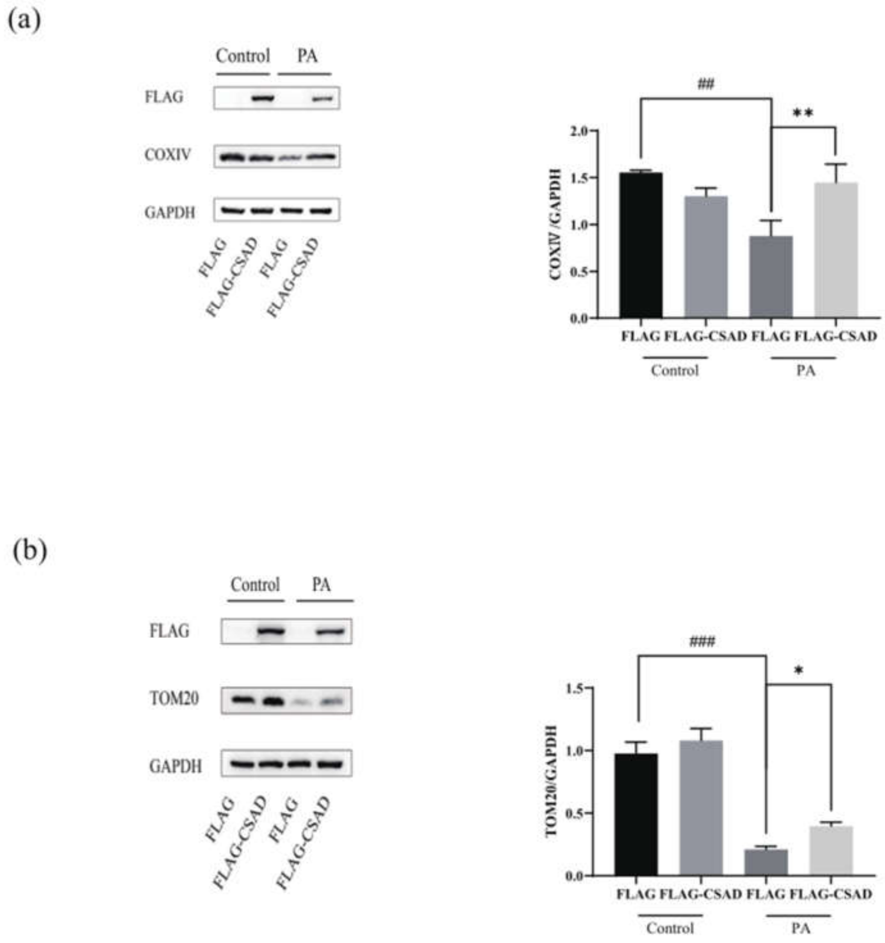

Supplementary Figure S3. Effect of CSAD on mitochondria-related protein in PA-treated L02 cells**.** The levels of COX-IV(**a**) and TOM20(**b**) in L02 cells. After transfected with FLAG or FLAG-CSAD for 48h, the L02 cells were incubated with or without 0.5mM PA for 24h, the protein levels of TOM20 and COX-IV were detected by Western blot and quantitative analysis using GAPDH as the normalizer. ##P<0.01, ###P<0.001*vs* FLAG group in control. *P< 0.05, **P<0.01*vs* FLAG group in PA.
